# NMDA receptor antagonist induced c-Fos expression in the medial entorhinal cortex during postnatal development

**DOI:** 10.3389/fncir.2025.1619534

**Published:** 2025-07-29

**Authors:** Feng Liang, Hong Wang, Robert Konrad Naumann

**Affiliations:** ^1^The Institute of Biomedical and Health Engineering, Shenzhen Institutes of Advanced Technology, Chinese Academy of Sciences, Shenzhen, China; ^2^Shenzhen-Hong Kong Institute of Brain Science-Shenzhen Fundamental Research Institutions, Shenzhen, China

**Keywords:** NMDA receptor antagonist, entorhinal cortex, c-Fos, parvalbumin, MK-801

## Abstract

*N*-methyl-D-aspartate receptor (NMDAR) antagonists, including ketamine, phencyclidine (PCP), and dizocilpine (MK-801), are an important class of drugs that can produce antidepressant, hallucinogenic, dissociative, psychotomimetic, and anesthetic effects in humans and animal models. To understand the effects of NMDAR antagonists on the brain, it is essential to map their actions at cellular resolution. We quantified c-Fos expressing cells in the mouse telencephalon after systemic injection of the potent NMDAR antagonist MK-801 and found a 10-fold higher density of c-Fos in the medial entorhinal cortex (MEC) compared to other regions of the telencephalon. c-Fos density was high in layer 3 of the dorsal MEC but low in other parts of the MEC. Since previous studies have shown that parvalbumin (PV) staining shows a strong dorsal-ventral gradient in the MEC, we investigated the spatial correlation between c-Fos and PV staining. We classified PV neurons based on their level of immunoreactivity and found that high and medium PV neurons were positively correlated with c-Fos density, while low PV neurons were negatively correlated. To understand the temporal correlation of c-Fos and PV staining, we examined their expression patterns after MK-801 injections during postnatal development. PV expression emerged on postnatal day 12, preceding c-Fos expression, which emerged on postnatal day 16. Our results suggest that local circuits comprising specific subtypes of inhibitory and excitatory neurons are critical for generating a sustained neuronal response to NMDAR antagonists. Furthermore, a high density of PV neuron input may be a prerequisite for the induction of c-Fos expression observed in MEC principal neurons. This study contributes to our understanding of how the brain responds to NMDAR antagonists in the developing and adult brain and reveals cell types in the dorsal MEC that are highly sensitive to this class of drugs.

## Introduction

The *N*-methyl-D-aspartate receptor (NMDAR) is one of the major receptor types that mediates glutamatergic neurotransmission. NMDARs are ubiquitously expressed in the brain and possess several unique properties, such as high permeability to calcium ions and voltage-dependent block by magnesium ions (Hansen et al., 2021; Zhou and Tajima, 2023). NMDAR dysfunction is commonly observed in several brain disorders, including depression, schizophrenia, epilepsy, and neurodegenerative diseases, and drugs that interfere with NMDAR function are promising therapeutic targets (Olney et al., 1999; Ghasemi and Schachter, 2011; Liu et al., 2019; Wang et al., 2022; Hanson et al., 2024; Jiang et al., 2024). The most commonly studied NMDAR antagonists, such as ketamine, phencyclidine (PCP), and dizocilpine (MK-801) can produce a variety of effects ranging from antidepressant to dissociative and anesthetic action in patients and animal models (Johnson and Jones, 1990; Olney and Farber, 1995; Aleksandrova et al., 2017). While all three drugs target the same binding site in the channel pore, they do so with different affinities and have different side effects (Song et al., 2018; Hansen et al., 2021). Thus, ketamine has several medical uses, but similar to PCP, is also used as a recreative drug, while MK-801 is most commonly used in animal models, due to its high potency and long half-life (Lodge and Mercier, 2015; Janus et al., 2023). Physiological responses to systemic application of NMDAR antagonists include increased gamma oscillations (Pinault, 2008), reduced hippocampal activity (Morgan et al., 2014), and disruption of spatial encoding by hippocampal place cells (Masuda et al., 2023), which together may underlie subjective reports of hallucinations, “dissociation” of space and time, and “out of body” experiences. Intuitively, NMDAR antagonists should reduce neuronal activity since they interfere with excitatory signaling between neurons. However, it has also been observed that some populations of neurons become more active, which may result from NMDAR antagonists acting on inhibitory neurons, i.e., by reducing inhibitory output downstream neurons may become disinhibited (Lisman et al., 2008; Booker and Wyllie, 2021; Krystal et al., 2024). Thus, to obtain a comprehensive understanding of the effects of NMDAR antagonists on the brain, it is necessary to map the cell types and circuits that change their activity patterns across different brain regions, drug doses, and developmental stages.

Immediate early genes are commonly used for brain-wide mapping of cellular activity (Hughes and Dragunow, 1995). For example, c-Fos expression is upregulated in highly active neurons (Sagar et al., 1988) and thus can be used as a tool to investigate changes in cellular activity after drug administration. Several studies have surveyed immediate early gene expression after injection of NMDA receptor antagonists in different brain regions (Dragunow and Faull, 1990; Gass et al., 1993; Väisänen et al., 2004). They found that a few cortical regions show consistently high levels of c-Fos induction, such as the medial entorhinal cortex (MEC), cingulate cortex, and the retrosplenial cortex (Castrén et al., 1993; Väisänen et al., 1999; Väisänen et al., 2004). Interestingly, Masuda et al. (2023) reported an increase in firing rates in the MEC after ketamine injections, which may correlate with the high levels of immediate early gene expression. However, the relative strength of responses to NMDAR antagonists (i.e., the density of c-Fos-positive cells) in different brain regions and cell types remains largely unexplored. The MEC is a key brain region for understanding spatial cognition because it contains several cell types with well-described firing patterns, such as grid cells, border cells, and head direction cells (Moser et al., 2008). At the cellular level, reconciling neuronal activity, morphology, and gene expression patterns remains challenging, but the MEC also harbors a number of circuit features that are organized at a higher level, such as the cell clusters in layer 2 or modules of grid cells with different spatial activity patterns (Preston-Ferrer and Burgalossi, 2018; Naumann et al., 2018; Tukker et al., 2022). One particularly striking pattern of cellular organization is the gradient of parvalbumin (PV) staining observed from dorsal to ventral MEC (Fujimaru and Kosaka, 1996; Beed et al., 2013; Boccara et al., 2015; Kobro-Flatmoen and Witter, 2019; Bjerke et al., 2021). PV neurons are a major type of inhibitory interneurons that includes fast-spiking neurons, which have been proposed to mediate the disinhibitory effects of NMDAR antagonists (Lisman et al., 2008; Aleksandrova et al., 2017; Krystal et al., 2024). However, the relation between the PV gradient and the pattern of cells activated by NMDAR antagonists in MEC remains unknown.

In addition to their beneficial effects in treating brain disorders, NMDAR antagonists can also have serious adverse health effects (Morgan et al., 2014; Lodge and Mercier, 2015). Therefore, it is critical to evaluate their effects not only in the adult brain, but also during different stages of development. For example, in adolescents, ketamine is considered as a promising treatment for depression, but it is also a common drug of abuse (Bokor and Anderson, 2014; Di Vincenzo et al., 2021). Interestingly, NMDAR antagonists can cause cellular damage and death in the brain during some stages of brain development, but remain innocuous during others (Olney et al., 1989; Sharp et al., 1991; Farber et al., 1995; Ikonomidou et al., 1999). However, studies investigating the c-Fos expression pattern of specific brain regions and cell types by NMDAR antagonists during development remain scarce (Jacobs et al., 2000; Pešić et al., 2010; Inta et al., 2017), and to our knowledge, none have examined the entorhinal cortex. Thus, here we aim to: (1) Compare cell densities of c-Fos-positive cells after systemic injection of the NMDAR antagonist MK-801 in the mouse telencephalon. (2) Describe the laminar and regional distribution of c-Fos-positive cells in the medial entorhinal cortex. (3) Determine the spatial correlation of the gradients of c-Fos and PV-positive cells in the medial entorhinal cortex and extend these observations to the developing mouse brain.

## Materials and methods

### Animals

Experimental procedures were performed according to guidelines of the Animal Care and Use Committees at the Shenzhen Institute of Advanced Technology (SIAT), Chinese Academy of Sciences (CAS), China (permit number SIAT-IACUC-250410-YGS-ROBERT NAUMANN-A2941). Male and female C57BL/6J mice were obtained from Beijing Vital River Laboratory Animal Technology Co., Ltd. Animals were group housed, food and water were given *ad libitum* and animals were kept on a light/dark cycle (light on at 7 a.m. and off at 7 p.m.). Pups were obtained on postnatal day P4, P8, P12, P14, P16, P20, P24, and P28, with P0 defined as the day of birth. A total of 10 mouse dams were used in this study. Adult animals were used between P60 and P80. MK-801 (MCE, HY-15084) was dissolved in sterile isotonic saline. Working solutions were freshly prepared before each experiment. A fixed volume/weight of 10 μL/g was used for each mouse. Adult mice were injected intraperitoneally with doses of 0.2, 0.5, 1, or 5 mg/kg MK-801 and allowed to survive for 4 h. Postnatal mouse pups were injected with 1 mg/kg MK-801 and sacrificed after 4 h. Control animals received an equal volume of 0.9% saline and were also sacrificed 4 h after injection.

### Tissue preparation

Animals were anesthetized by an intraperitoneal injection of 1% pentobarbital sodium, and then perfused transcardially with first 0.02 M phosphate buffered saline (PBS), followed by 4% formaldehyde, from paraformaldehyde, in 0.02 M phosphate buffered saline (PFA). After perfusion, brains were removed from the skull and postfixed in PFA overnight. Brains were then immersed in 30% sucrose solution in PBS for at least one night for cryoprotection. The brains were mounted on a freezing microtome to obtain 50 μm thick horizontal, coronal, sagittal or tangential sections (Naumann et al., 2016).

### Immunohistochemistry

Prior to the application of primary antibodies, sections were blocked in 5% BSA in PBS for 1 h at room temperature. Sections were transferred to primary antibody solution prepared in 1% BSA in 0.5% Triton X-100/PBS for 16–20 h at 4°C. Next, slices were washed in PBS three times for 20 min and transferred to secondary antibody solution at room temperature for at least 2 h or at 4°C overnight. After incubation and three times washes in PBS, slices were mounted on glass slides and coverslipped using Fluoromount (Sigma–Aldrich) mounting medium. The following primary antibodies were used (for details see Table 1): rabbit anti-c-Fos, mouse anti-c-Fos, mouse anti-PV (Parvalbumin), and rabbit anti-PCP4 (Purkinje cell protein 4). The mouse and rabbit anti c-Fos antibodies showed a highly similar staining pattern. The following secondary antibodies were used at a dilution of 1:1000: Alexa fluor 488 goat anti-rabbit (111-545-003, Jackson ImmunoResearch, RRID:AB_2338046) and Cy3 AffiniPure Donkey Anti-Mouse (715-165-150, Jackson ImmunoResearch, RRID:AB_2340813).

**TABLE 1:** Primary antibodies.

Name	Species	Cat.	RRID	Source	Supplier	Dilution
c-Fos	Rabbit monoclonal	2250	RRID:AB_2247211	Rabbit serum	Cell Signaling Technology	1:1000
c-Fos	Mouse monoclonal	66590-1-Ig	RRID:AB_2934481	Mouse serum	Proteintech	1:1000
PV	Mouse monoclonal	235	RRID:AB_10000343	Hybridoma cell supernatant	Swant	1:1000
PCP4	Rabbit polyclonal	HPA005792	RRID:AB_1855086	Rabbit serum	Sigma	1:1000

### Image acquisition

Images were acquired using an OLYMPUS BX61VS (VS120-S6-W) scanner (Olympus, Shinjuku-ku, Tokyo, Japan) and ZEISS LSM880 confocal microscope (ZEISS, Oberkochen, Germany). The fluorescent images were acquired in monochrome and color maps were applied to the images post acquisition. *Post hoc* linear brightness and contrast adjustment were applied uniformly to the image under analysis.

### Cell density and fluorescence quantification

For quantification of cell densities, six complete series of sections were generated, one of which was chosen randomly for quantification. Designation of brain regions and layers is based on the atlas of Paxinos and Franklin (2019), except for the entorhinal cortex, which we divide into medial and lateral parts (Witter et al., 2017). Images were opened in ImageJ software (National Institutes of Health, Bethesda, Maryland, United States)^1^. We measured the area of brain regions and layers, then counted cells manually, and expressed their density as cells/mm^2^. For each telencephalic region with detectable expression of c-Fos, we measured the cell density in at least three sections spaced at equal distances in three (coronal sections) or five (horizontal sections) brains. For more detailed density measurements in the medial entorhinal cortex, we counted cells four horizontal sections in the dorsal part (dMEC) and four horizontal sections in the ventral part of the medial entorhinal cortex (vMEC). To analyze the fluorescence intensity of PV or c-Fos staining, we selected three sagittal sections from each hemisphere and measured fluorescence values in 2 mm long stretches using the “plot profile” function in ImageJ. Fluorescence values were averaged every 100 μm, normalized to the highest value for each section, and then averaged across sections to obtain a profile of fluorescence intensity along the dorsal-ventral axis. Fluorescence intensity in individual neurons was measured using the “mean gray value” function in ImageJ and then normalized to the highest value.

### Statistics

Statistical testing was performed using Prism software (GraphPad Software Inc., V10, San Diego, CA, United States). No statistical method was used to predetermine sample size. Animals were randomly assigned to groups. In all figures, data are presented as mean ± SEM. The number of animals is indicated as *n*. Data were analyzed using Student’s *t*-test, ANOVA, or the Spearman correlation coefficient to compare the similarity of fluorescence and cell number profiles along the dorsal-ventral axis. Significance was assigned as follows: a = 0.05 (n.s. *p* > 0.05, *p < 0.05, ^**^p < 0.01, ^***^p < 0.001, ^****^p < 0.0001).

## Results

### Telencephalic regions expressing c-Fos after MK-801 injection

**FIGURE 1:**
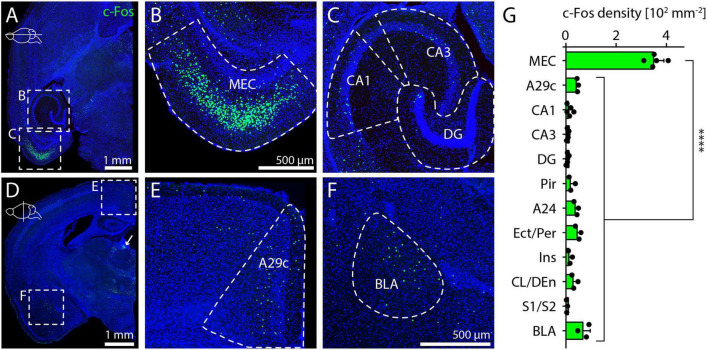
Telencephalic regions expressing c-Fos after MK-801 injection. **(A)** Overview image showing c-Fos staining in a horizontal section after injection of 1 mg/kg MK-801. Boxes indicate position of magnified images in **(B,C)**. **(B)** Example image of c-Fos staining in MEC after injection of 1 mg/kg MK-801. **(C)** Example image of c-Fos staining in the hippocampus after injection of 1 mg/kg MK-801. **(D)** Overview image showing c-Fos staining in a coronal section after injection of 1 mg/kg MK-801. White arrow indicates c-Fos staining in the lateral habenula. Boxes indicate position of magnified images in **(E,F)**. **(E)** Example image for c-Fos staining in A29c after injection of 1 mg/kg MK-801. **(F)** Example image for c-Fos staining in BLA after injection of 1 mg/kg MK-801. **(G)** c-Fos density in different regions of the telencephalon. Data from horizontal sections for MEC, CA1, CA3, and DG (*N* = 5) and coronal sections for all other brain regions (*N* = 3). One-way ANOVA (*p* < 0.0001) followed by Dunnett’s post-hoc test, ****p < 0.0001. MEC, medial entorhinal cortex; CA1/3, cornu ammonis 1/3; DG, dentate gyrus; A29c, cingulate cortex area 29c; BLA, basolateral amygdala; Pir, piriform cortex; A24, cingulate cortex area 24; Ect/Per, ectorhinal cortex/perirhinal cortex; Ins, insular cortex; CL/DEn, claustrum/dorsal endopiriform nucleus; S1/S2, primary/secondary somatosensory cortex.

To assess which brain regions and cell types are activated by NMDAR antagonists, we selected the potent antagonist MK-801 at a dose of 1 mg/kg and sacrificed animals 4 h after intraperitoneal drug injection (Väisänen et al., 1999). Subsequently, we surveyed c-Fos immunostaining in the whole mouse brain in series of coronal or horizontal sections, but focus only on the telencephalon in this study. Consistent with previous studies (Väisänen et al., 1999; Väisänen et al., 2004), we observed a high density of c-Fos positive cells in the medial entorhinal cortex (MEC), but not in the hippocampus (Figures 1A–C). In midline cortical regions (A29c, A24) and the basolateral amygdala (BLA), c-Fos density was somewhat higher than in adjacent regions (Figures 1D–F). In summary, the average density of c-Fos was 355 cells per mm^2^ in the MEC, approximately 10-fold higher than on average in other regions of the telencephalon with detectable c-Fos expression (Figure 1G). In addition, we also observed c-Fos expression in subcortical regions, including the lateral habenula (Figure 1D, white arrow), the pre-Edinger-Westphal nucleus, and several other regions such as the paraventricular and reuniens nuclei of the thalamus as described previously (Dragunow and Faull, 1990; Gass et al., 1993; Väisänen et al., 2004).

### Layer-specific c-Fos expression and dorsal-ventral gradient in medial entorhinal cortex

**FIGURE 2:**
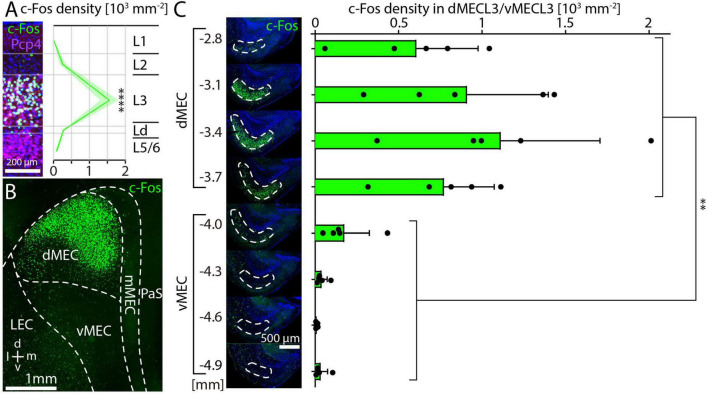
Layer-specific c-Fos expression after MK-801 injection and dorsal-ventral gradient in medial entorhinal cortex. **(A)** c-Fos and PCP4 staining in columns of dorsal medial entorhinal cortex (*N* = 5). c-Fos density was measured for each layer. The solid line and shaded area represent the mean and SEM, respectively. One-way ANOVA (*p* < 0.0001) followed by Dunnett’s post-hoc test, ****p < 0.0001. **(B)** Tangential section showing dense c-Fos staining in the dorsal part of MEC. **(C)** c-Fos density measured in layer 3 of medial entorhinal cortex (*N* = 5). Unpaired *t*-test **p = 0.001.

Consistent with previous results (Väisänen et al., 1999), we found the highest density of c-Fos-positive cells in layer 3 of the MEC. Here, we quantified this result by measuring cell density across layers in 250 μm-wide columns obtained from horizontal sections with strong c-Fos expression and found an average density of 1,546 cells per mm^2^ in layer 3, significantly higher than in other layers (Figure 2A). To confirm the position of c-Fos cells in layer 3, we co-stained sections for c-Fos and PCP4 (Purkinje cell protein 4), a marker for layer 3 and layer 5 MEC principal neurons (Figure 2A; Ohara et al., 2021). We counted c-Fos and PCP4 cell numbers in layer 3 of dMEC and found that 98.5% (1,110/1,127) of c-Fos-positive cells were also PCP4-positive and that 82.5% (1,110/1,345) of the PCP4-positive cells were also c-Fos-positive, suggesting that c-Fos is expressed almost exclusively layer 3 principal cells. The subset of PCP4-positive cells that did not express c-Fos were mainly located in the deeper parts of layer 3. To better understand the topography of c-Fos expression in MEC, we prepared tangential sections (Naumann et al., 2016; Ray et al., 2017), where c-Fos expression is visible in a restricted region in the dorsal part of MEC (Figure 2B). Based on this result, we provisionally divided the MEC into three subregions. The dorsal part of the MEC (dMEC) contained the vast majority of c-Fos positive cells, while a thin medial region (mMEC) and the ventral part (vMEC) contained very few c-Fos positive cells (Figure 2B). To quantify this observation, we prepared horizontal sections and measured c-Fos density in dMEC and vMEC. Average c-Fos density was 850 cells per mm^2^ in layer 3 of dMEC and 64 cells per mm^2^ in layer 3 of vMEC, and thus more than 10-fold higher in dMEC than in vMEC (Figure 2C).

### c-Fos expression in medial entorhinal cortex is correlated with dose of MK-801

**FIGURE 3:**
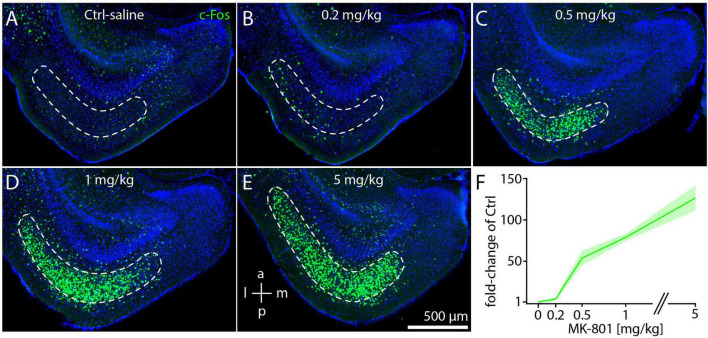
c-Fos expression in medial entorhinal cortex is correlated with dose of MK-801. **(A–E)** Example image of c-Fos staining in saline-injected control animals (Ctrl) or after injection of 0.2 mg/kg **(B)**, 0.5 mg/kg **(C)**, 1 mg/kg **(D)**, and 5 mg/kg **(E)** MK-801 (*N* = 3 for each dose). **(F)** Densities of c-Fos cells at different doses shown as fold-changes relative to saline-injected controls. The solid line and shaded area represent the mean and SEM, respectively.

We also investigated whether c-Fos expression was ose-dependent after injection of MK-801 d (Väisänen et al., 1999). By measuring cell density in horizontal sections as shown in Figure 2C, we found that a dose of 0.2 mg/kg leads to a small increase of c-Fos staining in layer 3 of dMEC compared to controls (Figures 3A, B). However, the major shift in c-Fos staining occurs at a dose of 0.5 mg/kg (Figure 3C). Further increases in MK-801 dose led to further, but relatively smaller increases in c-Fos density (Figures 3D, E). In summary, c-Fos expression in response to MK-801 undergoes a marked transition between doses of 0.2 and 0.5 mg/kg, which induce 4-fold and 54-fold increases in c-Fos density compared to controls, respectively (Figure 3F). Doses of 1 mg/kg and 5 mg/kg MK-801 induced 79-fold and 126-fold increases in c-Fos density compared to controls (Figure 3F).

### Parvalbumin and c-Fos fluorescence along the dorsal-ventral axis in MEC

**FIGURE 4:**
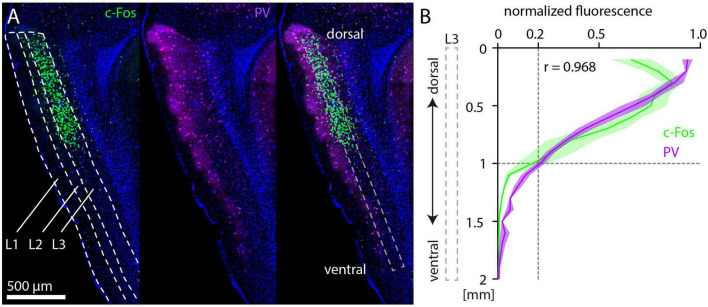
Parvalbumin and c-Fos fluorescence after MK-801 injection along the dorsal-ventral axis in medial entorhinal cortex (MEC). **(A)** Example image of c-Fos (left) and parvalbumin (PV) (middle) staining. The overlay (right) also indicates the window for measurement of fluorescence in layer 3 of MEC. **(B)** Normalized c-Fos and PV fluorescence measured in layer 3 of MEC from 12 sagittal sections (three sections per hemisphere, *N* = 2). The solid line and shaded area represent the mean and SEM, respectively. r indicates the Spearman correlation coefficient.

Parvalbumin antibody staining shows a striking intensity gradient from dorsal (high) to ventral (low) in the MEC, which is readily apparent in sagittal sections (Beed et al., 2013; Boccara et al., 2015). Since PV neurons are likely to be important for the disinhibitory effects of NMDAR antagonists (Lisman et al., 2008; Aleksandrova et al., 2017; Krystal et al., 2024), we wondered whether the gradient of PV fluorescence is spatially correlated with c-Fos expression after MK-801 injection. To test this, we prepared sagittal sections of mouse brains after injection of 1 mg/kg MK-801 and performed immunostaining against c-Fos and PV (Figure 4A). PV staining results were qualitatively similar to previous studies, indicating that MK-801 did not alter the dorsal-ventral gradient of PV expression. We measured fluorescence intensity in layer 3 of MEC along the dorsal-ventral axis and averaged values every 100 μm. Normalized fluorescence values for PV and c-Fos were strikingly similar (Figure 4B), although c-Fos fluorescence was on average weaker in the most dorsal part of MEC. Both curves dropped to approximately 20% of the maximum at a distance of 1 mm from the dorsal border of MEC.

### Parvalbumin and c-Fos cell density along the dorsal-ventral axis in MEC

**FIGURE 5:**
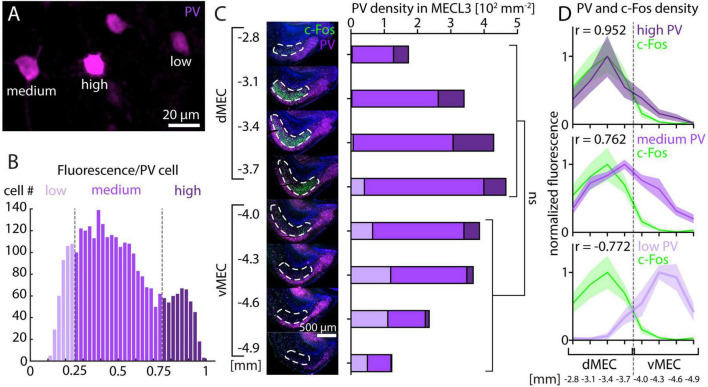
Parvalbumin and c-Fos cell density after MK-801 injection along the dorsal-ventral axis in medial entorhinal cortex (MEC). **(A)** Example image showing parvalbumin (PV) neurons with different levels of fluorescence intensity. **(B)** Histogram of fluorescence intensity for PV neurons from layer 3 of MEC. Low PV neurons are defined as having 25% or less of maximum fluorescence intensity. Medium PV neurons are defined as having between 25% and 75% of maximum fluorescence intensity. High PV neurons are defined as having 75% or more of maximum fluorescence intensity. **(C)** The density of PV neurons measured in layer 3 of medial entorhinal cortex (*N* = 5) is not significantly different between dMEC and vMEC, unpaired *t*-test *p* = 0.1456. The densities of high, medium, and low PV neurons are significantly different between dMEC and vMEC, unpaired *t*-test *p* = 0.0195 (high PV), *p* = 0.0199 (medium PV), *p* = 0.0006 (low PV). **(D)** Normalized c-Fos and PV neuron density measured in layer 3 of MEC is highly correlated for high PV neurons and c-Fos and negatively correlated for low PV neurons and c-Fos (*N* = 5). The solid line and shaded area represent the mean and SEM, respectively.

While overall PV fluorescence clearly decreases along the dorsal-ventral axis of MEC, previous studies have found either very small (Beed et al., 2013) or more steep decreases in PV neuron density (Fujimaru and Kosaka, 1996; Kobro-Flatmoen and Witter, 2019). Thus, it remains unclear if changes in PV neuron density can fully explain the observed PV fluorescence gradient. We therefore asked whether PV neurons with different levels of fluorescence intensity are differentially distributed along the dorsal-ventral axis of the MEC. As previously described for the hippocampus (Donato et al., 2013), we found that PV neurons in the MEC can be subdivided into different subsets based on their level of PV immunoreactivity (Figure 5A). In the same animals and sections shown in Figure 2, we measured fluorescence intensity of 2,906 PV neurons in the MEC and divided them into three groups comprising the upper (high PV) and lower (low PV) 25% of fluorescence intensity and the remaining intermediate (medium PV) group (Figure 5B). Total PV neuron density was on average higher in dMEC than in vMEC, but this difference was not significant (Figure 5C). However, the densities of high, medium, and low PV neurons show significant differences between dMEC and vMEC. High PV neurons were more prominent and low PV neurons were almost absent in dMEC, while the relation was reversed in vMEC (Figure 5C). Consequently, we found a high correlation of high PV and c-Fos density and a negative correlation of low PV and c-Fos density (Figure 5D). Interestingly, we did not detect any PV neurons that were also c-Fos positive.

### Postnatal development of c-Fos and PV in dorsal MECL3 after MK801

**FIGURE 6:**
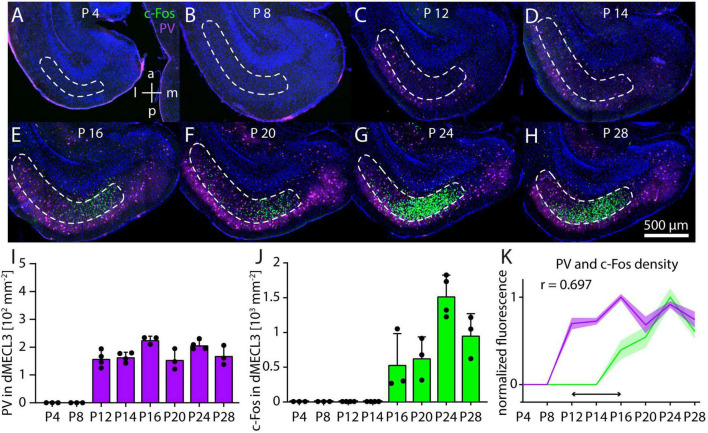
Postnatal development of c-Fos and parvalbumin (PV) in dorsal MECL3 after MK801. **(A–H)** Example image of c-Fos staining after injection of 1 mg/kg MK-801 on postnatal day 4 (*N* = 3) **(A)**, postnatal day 8 (*N* = 3) **(B)**, postnatal day 12 (*N* = 4) **(C)**, postnatal day 14 (*N* = 4) **(D)**, postnatal day 16 (*N* = 3) **(E)**, postnatal day 20 (*N* = 3) **(F)**, postnatal day 24 (*N* = 4) **(G)**, and postnatal day 28 (*N* = 3) **(H)**. **(I)** Density of PV neurons in layer 3 of dorsal MEC in postnatal development. **(J)** Density of c-Fos in layer 3 of dorsal MEC in postnatal development. **(K)** Normalized densities of c-Fos and PV neurons in postnatal development. Horizontal arrow indicates delay between PV and c-Fos expression. The solid line and shaded area represent the mean and SEM, respectively.

So far, it was unknown at which point in postnatal development c-Fos immunoreactivity emerges in response to MK-801 in the MEC. We combined c-Fos and PV immunostaining in horizontal sections of the developing mouse brain and measured cell densities in layer 3 of dMEC (dMECL3) after injection of 1 mg/kg MK-801 in the pups using the same conditions as for the experiments in adult mice. On postnatal day 4 (P4) and P8, we observed neither c-Fos nor PV staining in dMECL3 (Figures 6A, B). On P12 and P14 PV cells became detectable (Figures 6C, D). On P16 and P20 we first observed c-Fos staining after MK-801 injection (Figures 6E, F). Interestingly, c-Fos staining was not uniform but concentrated in the medial part of dMECL3, whereas PV neurons were evenly distributed in all parts of layer 3. On P24 and P28, c-Fos and PV staining became more dense and increasingly similar to the pattern observed in adult animals (Figures 6G, H). Thus, during postnatal development PV staining is first detectable in layer 3 of MEC on P12 (Figure 6I). The density of PV neurons was stable, but we did not distinguish PV neurons based on their intensity as in adult animals. Overall, it appears that PV staining intensity is increasing during the developmental period observed, indicating ongoing maturation. In contrast, c-Fos staining after MK-801 injection first appears on P16, possibly with a transient peak of c-Fos density on P24 (Figure 6J). In summary, we found that the onset c-Fos staining has a delay of at least 4 days relative to PV staining after MK-801 injection (Figure 6K).

## Discussion

Using immunostaining in the adult and developing mouse brain, we quantitatively compared the effect of the non-competitive NMDAR antagonist MK-801 on c-Fos expression in different regions of the telencephalon. We found the highest level of c-Fos expression in layer 3 of the dorsal MEC (Figures 1, 2). Antibody staining revealed that the most dramatic change in the c-Fos staining pattern occurs at a dose of 0.5 mg/kg MK-801, although higher doses still induce further increases in c-Fos density (Figure 3). PV neurons have a high density in the dorsal MEC and are thought to be critical for mediating the brain’s response to NMDAR antagonists. Our results show a high spatial correlation of PV and c-Fos expression in the adult MEC (Figures 4, 5), while PV expression precedes c-Fos expression in by several days during postnatal development (Figure 6). These observations extend previous studies (Väisänen et al., 1999; Väisänen et al., 2004) and suggest that two specific cell types, PV neurons and principal neurons in layer 3 of the dorsal MEC, play a key role in generating the major response to NMDAR antagonists in the adult and developing telencephalon.

### Structure and function of layer 3 in the medial entorhinal cortex

The entorhinal cortex is the key communication node between the neocortex and the hippocampus. The primary role of the medial entorhinal cortex is to format spatial information about the environment for further processing in the hippocampus, but it may have a much broader function in cognition (Dong and Fiete, 2024). Collectively, these functions depend on grid cells and other spatially selective cell types located mainly in layer 2 of the medial entorhinal cortex (Moser et al., 2008). In contrast, layer 3 has been referred to as the “unknown land” of the medial entorhinal cortex (Witter et al., 2017), as its circuits and structure-function relationships are much less understood than those of other layers. The structure of the medial entorhinal cortex has been reviewed in detail (Insausti, 1993; Witter et al., 2017; Naumann et al., 2018; Kobro-Flatmoen and Witter, 2019; Tukker et al., 2022). In brief, some of the key aspects of MEC layer 3 structure and function are: (1) Layer 3 principal (excitatory) neurons in the MEC are thought to form a homogeneous cell population (Tang et al., 2015; Tukker et al., 2022; Kanter et al., 2025). (2) In rats, monkeys, and humans, layer 3 contains two to three times more neurons than layer 2 (Amaral et al., 2025). (3) Layer 3 sends topographically organized projections to the CA1 and subiculum regions of the hippocampus (Honda et al., 2012; Igarashi et al., 2014). (4) Under anesthesia and during sleep, layer 3 shows prominent slow oscillations (Hahn et al., 2012; Beed et al., 2020; Haam et al., 2023). (5) Layer 3 neurons are important for temporal association memory (Suh et al., 2011), provide an instructive signal to CA1 during spatial learning (Grienberger and Magee, 2022), and contain a high density of predictive grid cells encoding information about future goal locations (Ouchi and Fujisawa, 2024). (6) Layer 3 neurons may be affected in epilepsy, Alzheimer’s disease, and schizophrenia (Kanter et al., 2025). In summary, so far very little is known about regional differences of layer 3 principal neurons in the MEC that could explain the high density of c-Fos staining in the dorsal MEC after MK-801 injection.

### Parvalbumin gradients in the adult medial entorhinal cortex

Parvalbumin neurons provide high-powered inhibitory input to local principal neurons (Hu et al., 2014). However, when affected by NMDAR antagonists, PV neurons reduce their activity and thus disinhibit their target neurons (Lisman et al., 2008; Aleksandrova et al., 2017; Krystal et al., 2024), which may explain the high levels of c-Fos staining observed in response to NMDAR antagonists in subsets of principal neurons. Because PV neurons are widely distributed in the cortex and other brain regions, the question remains why this disinhibitory effect is only observed in some parts of the brain. In particular, it is unclear whether disinhibition mediated by PV neurons can explain why principal neurons in layer 3 of the dorsal MEC are activated. One possible reason is that a high density of PV inputs is required for NMDAR antagonist-induced increases in neuronal activity. Indeed, numerous studies have described a high density of PV staining in the dorsal MEC and a low density in the ventral MEC (Fujimaru and Kosaka, 1996; Beed et al., 2013; Boccara et al., 2015; Booth et al., 2016; Kobro-Flatmoen and Witter, 2019; Grosser et al., 2021; Bjerke et al., 2021). While most of these studies have focused on the gradient of PV staining in layer 2 of the MEC, a similar, or perhaps even steeper gradient is apparent in layer 3 (Figure 4; Beed et al., 2013; Booth et al., 2016; Bjerke et al., 2021). The differences in fluorescence intensity could be explained by a higher density of PV neurons in the dorsal MEC (Fujimaru and Kosaka, 1996; Kobro-Flatmoen and Witter, 2019). However, the results of Beed et al. (2013) suggest that the differences in PV neuron density are minimal. Our results show that neurons with high- and medium-PV intensity in layer 3 do indeed have a higher density in the dorsal MEC, but the difference is not significant when low-PV intensity neurons are included (Figure 5), which may explain the conflicting results of previous studies.

### Parvalbumin neuron subtypes in the adult medial entorhinal cortex

Surprisingly, Grosser et al. (2021) recently found that PV neurons show minimal differences in dendritic morphology and intrinsic physiology between dorsal and ventral MEC. Although axons were shorter in dorsal MEC PV neurons, they had a significantly higher density of boutons (Grosser et al., 2021), which may account for the differences in fluorescence intensity observed along the dorsal-ventral axis with PV antibody staining. An alternative to a mere high density of PV inputs being necessary for NMDAR antagonist-induced increases in neuronal activity would be if there were specific subtypes of PV neurons that target layer 3 principal neurons in dMEC. Interestingly, Gurgenidze et al. (2022) recently described a subset of PV neurons in MEC with a distinct “stuttering” firing pattern and a preference for axonal distribution in layer 3. However, the response of this PV neuron subtype to NMDAR antagonists and whether it is present specifically in the dMEC remains unknown. Acute or chronic injections of NMDAR antagonists can alter PV expression levels or the number of PV neurons or boutons (Honeycutt and Chrobak, 2018). However, the PV gradient we observed after MK-801 injection appears similar to the gradient observed without drug injection (Fujimaru and Kosaka, 1996; Beed et al., 2013) or in a mouse model of Alzheimer’s disease (Booth et al., 2016). Instead, we focused on the question whether the PV gradient spatially correlates with c-Fos expression in layer 3 of MEC after MK-801 injection. Based on these earlier findings and our observations (Figures 4, 5), we conclude that a high density of PV neurons and consequently, PV innervation is likely to be a prerequisite for a strong activation of MEC principal neurons by NMDAR antagonists.

### Development of parvalbumin- and c-Fos expression in response to NMDAR antagonists

These results prompted us to investigate the response to NMDAR antagonists in the MEC during early postnatal development. Some previous studies have addressed this question in other brain areas (Jacobs et al., 2000; Pešić et al., 2010; Inta et al., 2017), but to our knowledge none have focused on the MEC. While we focused on the medial entorhinal cortex, we did not observe a prominent transient change in hippocampal c-Fos expression after MK-801 injection during postnatal development, as observed in a previous study (Inta et al., 2017). This may be due to differences in experimental conditions. However, we did observe peak in c-Fos expression in MEC on postnatal day 24, as reported by Inta et al. (2017) for the hippocampus. In contrast, the postnatal development of PV neurons in MEC has received considerable attention (Couey et al., 2013; Lensjø et al., 2017; Donato et al., 2017; Berggaard et al., 2018). While most of the latter studies have focused on layer 2 of the MEC, a common conclusion is that PV staining in the MEC first appears a few days before eye opening (postnatal day 14), but critical steps of PV neuron maturation continue for several days thereafter. If a high density of functional PV neurons is a prerequisite for an adult-like response to NMDAR antagonist injections, then this response should occur only after the maturation of PV neurons. This is indeed what we observed (Figure 6). Interestingly, we found the earliest c-Fos staining on P16, which is also when grid cells begin to exhibit hexagonal activity patterns (Wills et al., 2010; Langston et al., 2010), and when PV neurons start to be ensheathed by perineuronal nets (Lensjø et al., 2017), although both grid cell- and PV neuron maturation continues over the following days. We also observed that c-Fos staining after MK-801 injection is initially concentrated on the medial side of the dorsal MEC, but it remains unknown whether PV neuron maturation also follows a medial to lateral pattern. Recent studies have emphasized the dorsal to ventral maturation pattern of the MEC (Ray and Brecht, 2016; Donato et al., 2017; Berggaard et al., 2018). However, the main difference in c-Fos staining that we observe between dorsal and ventral MEC persists throughout all stages of development, suggesting instead that dMEC and vMEC are functionally and anatomically distinct compartments in the present context, rather than showing gradual differences. Conceivably, changes in the expression of NMDARs during postnatal development could influence the activation of MEC neurons by NMDAR antagonists. At the level of the whole brain, there is some evidence for changes in NMDAR subunit expression during postnatal development. For example, NR1 and NR2B subunit expression remains relatively constant, whereas expression of the NR3 subunit decreases and the expression of the NR2A subunit increases during postnatal development (Monyer et al., 1994; Sheng et al., 1994; Wong et al., 2002). However, developmental changes in NMDAR subunit expression vary by brain region (Murillo et al., 2020) and data focusing on the entorhinal cortex remain scarce. Furthermore, broad changes in cortical NMDAR subunit expression do not readily explain the sudden increase in c-Fos expression after NMDAR antagonist injection observed between P12 and P16. Interestingly, fast spiking neurons in the rat prefrontal cortex show a substantial decrease in NR2B subunits during postnatal development (Wang and Gao, 2009), indicating that developmental changes in subunit expression differ between cell types. In the future, cell type-specific information about NMDAR subunit expression and composition during MEC development may contribute to understanding the abrupt onset of responses to NMDAR antagonists.

### Retrosplenial cortex function and cellular damage in response to NMDAR antagonists

Systemic injections of NMDAR antagonists have been shown to induce substantial amounts of cell death in several cortical regions in on postnatal day 7, but not at later stages of development (Ikonomidou et al., 1999). An earlier study had also shown that MK-801 induces cellular damage specifically in the retrosplenial cortex, but only in adult and young adult animals (Farber et al., 1995; Olney et al., 1989). These studies suggest that there may be a period in postnatal development when animals are resistant to the adverse effects of NMDAR antagonists. However, the relation of high levels cellular activity and cellular degeneration remains poorly understood. Väisänen et al. (1999) have shown that injection of 5 mg/kg MK-801, 15 mg/kg phencyclidine (PCP), and 50 mg/kg ketamine result in comparable c-Fos expression in the MEC. Interestingly, this dose of ketamine induces a dissociative behavioral effect, that may be mediated by neuronal circuits in the retrosplenial cortex (Vesuna et al., 2020; Hu et al., 2025). The retrosplenial cortex projects to the dorsal - but not the ventral - part of MEC (Burwell and Amaral, 1998; Jones and Witter, 2007; Dubanet and Higley, 2024; Kanter et al., 2025). Thus, further studies of the circuits involving the retrosplenial cortex and the dorsal MEC are necessary to understand the neural mechanisms of dissociative behavior. In particular, identifying, characterizing, and manipulating the activity of PV neurons in these brain regions appears to be a promising route to understand the cognitive effects of NMDAR antagonists (Li et al., 2002; Hu et al., 2025).

## Conclusion

We examined c-Fos expression in the MEC in response to systemic administration of NMDAR antagonists (Väisänen et al., 1999; Väisänen et al., 2004) and discovered a high correlation with the local gradient of PV expression (Fujimaru and Kosaka, 1996; Beed et al., 2013; Kobro-Flatmoen and Witter, 2019; Bjerke et al., 2021). Thus, a high density of inputs from PV neurons may be a prerequisite for the sustained c-Fos expression we observed in layer 3 principal neurons. This is supported by our observation that during postnatal development, c-Fos expression does not appear until several days after local PV expression emerges, presumably when PV neurons have attained a sufficient state of maturation (Lensjø et al., 2017). However, high levels of PV staining are not exclusive to the MEC and even there, the density of PV staining is highest in layer 2. Similarly, other brain regions with dense PV staining, such as the parasubiculum, hippocampal CA3, layer 4 of the somatosensory cortex, and large parts of the thalamus have a low density of c-Fos positive cells. Therefore, other factors are likely to be important for explaining the pattern of c-Fos expression we observed. For example, the retrosplenial cortex may play an important role for understanding the dissociative effects ketamine (Vesuna et al., 2020; Hu et al., 2025) and thus it is striking that retrosplenial cortex projections to the MEC target the same area activated by NMDAR antagonists. Finally, and perhaps most importantly, because MEC layer 3 principal neurons are thought to constitute a homogeneous population (Tang et al., 2015; Tukker et al., 2022; Kanter et al., 2025), it is surprising that a simple injection of an NMDAR antagonist can selectively activate a specific subset of layer 3 neurons in the dorsal MEC, showing that the immediate early gene response is highly cell type specific (Yap and Greenberg, 2018). The differences between dorsal, ventral, and medial MEC layer 3 principal neurons are likely to be subtle and therefore particularly useful for identifying causal mechanisms for cell-type specific activity, vulnerability, or pathology (Kampmann, 2024). Our study contributes to this effort by identifying the correlation between PV staining and c-Fos expression in response to NMDAR antagonists and its emergence during postnatal development.

## Data Availability

The original contributions presented in this study are included in this article/supplementary material, further inquiries can be directed to the corresponding author.

## Footnotes

1
https://imagej.nih.gov/ij/
